# Changes of Infant- and Family-Centered Care Practices Administered to Extremely Preterm Infants During Implementation of the NIDCAP Program

**DOI:** 10.3389/fped.2021.718813

**Published:** 2021-09-03

**Authors:** Valérie Klein, Claire Zores-Koenig, Laurence Dillenseger, Claire Langlet, Benoît Escande, Dominique Astruc, Isabelle Le Ray, Pierre Kuhn, Strasbourg NIDCAP Study group

**Affiliations:** ^1^Service de Pédiatrie, Centre Hospitalier de Haguenau, Haguenau, France; ^2^Service de Médecine et Réanimation du Nouveau-né, Centre Hospitalier Universitaire de Strasbourg, Strasbourg, France; ^3^Service d'Obstétrique-Gynécologie, Centre Hospitalier Universitaire de Strasbourg, Strasbourg, France; ^4^Institut de Neurosciences Cellulaires et Intégratives, CNRS, Université de Strasbourg, Strasbourg, France

**Keywords:** developmental care, extremely preterm infant, family centered care, implementation, Neonatal Individualized Developmental Care and Assessment Program, pain management, parental involvement, skin-to-skin contact

## Abstract

**Introduction:** Many studies have evaluated the Neonatal Individualized Developmental Care and Assessment Program (NIDCAP), but few studies have assessed changes in infant- and family-centered developmental care (IFCDC) practices during its implementation.

**Objectives:** The primary objective of this single center study was to investigate the impact of the implementation of the NIDCAP program on IFCDC practices used for management of extremely preterm infants (EPIs). The secondary objective was to determine during implementation the impact of this program on the short-term medical outcomes of all EPIs hospitalized at our center.

**Methods:** All EPIs (<28 weeks gestational age) who were hospitalized at Strasbourg University Hospital from 2007 to 2014 were initially included. Outborn infants were excluded. The data of EPIs were compared for three time periods: 2007 to 2008 (pre-NIDCAP), 2010 to 2011, and 2013 to 2014 (during-NIDCAP implementation) using appropriate statistical tests. The clinical and caring procedures used during the first 14 days of life were analyzed, with a focus on components of individualized developmental care (NIDCAP observations), infant pain management (number of painful procedures, clinical pain assessment), skin-to-skin contact (SSC; frequency, day of initiation, and duration), and family access and involvement in the care of their children (duration of parental presence, parental participation in care). The short-term mortality and morbidity at discharge were evaluated.

**Results:** We examined 228 EPIs who received care during the three time periods. Over time, painful procedures decreased, but pain evaluations, parental involvement in care, individualized observations, and SSC increased (all *p* < 0.01). In addition, the first SSC was performed earlier (*p* = 0.03) and lasted longer (*p* < 0.01). There were no differences in mortality and morbidity, but there were reductions in the duration of mechanical ventilation (*p* = 0.02) and the time from birth to first extubation (*p* = 0.02), and an increase of weight gain at discharge (*p* = 0.02).

**Conclusion:** NIDCAP implementation was accompanied by progressive, measurable, and significant changes in IFCDC strategies. There were, concomitantly, moderate but statistically significant improvements in multiple important outcome measures of all hospitalized EPI.

## Introduction

Population-based studies in Europe reported that extremely preterm infants (EPIs) remain at high risk of neonatal morbidity and neurodevelopmental sequelae despite the development of medical interventions that improved their survival during the last decade (1–3). There is also evidence that EPIs experience long-term alterations of developmental trajectories and academic outcomes (4, 5).

Besides the well-identified medical causes of these adverse outcomes, these impairments are also partly attributable to the early sensory experiences of EPIs during critical periods of brain development (6, 7). The environment of the neonatal intensive care unit (NICU) is particularly important, because it has many atypical stimuli that are not adapted to their sensory abilities and expectations. In addition, their hospitalization is often associated with an early separation from the parents, so they often lack access to biologically meaningful and developmentally supportive stimuli (6, 8–10). Caregivers have therefore developed infant- and family-centered developmental care (IFCDC) programs that address these issues. These programs aim to adapt the hospital environment and support a family-centered and individualized approach based on the evaluation of the infant's cues and family needs. Their general goal is to improve the outcomes of this highly vulnerable population of infants (11).

This holistic philosophy of care for infants incorporates theories and concepts derived from studies in neurodevelopment and neuro-behavior, parent-infant interactions and parental involvement, and breastfeeding promotion. It is based on several developmental care interventions that can be used alone, or combined into more formalized developmental care programs. The Neonatal Individualized Developmental Care and Assessment Program (NIDCAP) adjusts interventions to the needs of each child and is a highly conceptualized intervention. Previous studies confirmed the NIDCAP provided short-term benefits (12, 13). A randomized controlled trial (RCT) that examined infants with a mean gestational age (GA) of 27 weeks reported improvement in neurodevelopment at 18 months of post-menstrual age, although the long-term effects of this program are still uncertain (14–17). Moreover, it appeared that the preterm infants who benefited most from IFCDC strategies had strong involvement of parents in single family rooms, were the most immature infants with GAs <29 weeks (18).

The NIDCAP is a complex intervention that contains several interacting components. It requires important behavioral changes in the health care team and parents for support, and it affects different organizational levels within the hospital. There are numerous outcomes and they may be variable (19). Thus, a key question in evaluating the NIDCAP, as for other complex interventions, is whether it is effective in everyday practice (19, 20). Observational studies can provide additional information on the efficacy of this intervention and are complementary to RCTs in evaluating the effectiveness of this complex intervention. Observational studies are crucial because implementation of NIDCAP is a long process that is challenging for neonatal teams (21), and it requires significant involvement of the entire team and strong institutional support (14, 22). In fact, the NIDCAP requires education and training of health care professional so they can function as competent NIDCAP-certified health care professionals. There is therefore a need for more research to evaluate different methods of NIDCAP implementation (23).

A nation-wide population-based study of NICUs in France showed that family access, the involvement of parents in child care, and early initiation of skin-to-skin contact (SSC) increased between 2004 and 2011, although there were variations among centers (24). The implementation of neurodevelopmental care practices at the level of individual centers increased due to specific neurodevelopmental care training and policies that support IFCDC. In particular, there is evidence that the NIDCAP had a positive impact on early initiation of SSC and breastfeeding for very preterm infants in neonatal centers in France (24). However, this study only evaluated two core developmental care measures, and did not report data regarding parental involvement and pain management. Moreover, there are few detailed studies of the impact of the NIDCAP on IFCDC practices for EPIs at the level of individual centers during the implementation of this program.

The Department of Neonatology at Strasbourg University Hospital started to implement the NIDCAP in 2008, making this institution well-suited for a study of the efficacy of this intervention. The primary objective of this study was to evaluate the impact of the NIDCAP on IFCDC clinical practices for EPIs, with a focus on infant pain management, SSC practices, and parental presence and involvement in the care of their newborn infants. The secondary objective was to determine whether this change in practices already affected the short-term morbidity and mortality observed in the whole population of EPIs who were hospitalized at our center.

## Materials and Methods

### Design and Study Population

This retrospective, single-center, comparative study examined the IFCDC strategies used for EPIs in the NICU of the Strasbourg University Hospital before (2007–2008) and during (2010–2011 and 2013–2014) implementation of the NIDCAP program. All included EPIs (GA <28 weeks) were from single or multiple pregnancies, had no malformations, and were born at Strasbourg University Hospital during the indicated study periods.

### Data Collection

The implementation of the NIDCAP was evaluated for EPIs who survived longer than 14 days. Three main IFCDC indicators were examined during the first 14 days of life for each EPI: (i) Exposure to the NIDCAP program (number of NIDCAP behavioral observations by NIDCAP-certified healthcare providers, and number of infants benefiting from NIDCAP interventions, based on two or more observations during the hospital stay); (ii) Pain management (number of painful procedures and number pain score evaluations); and (iii) Family-centered care practices and SSC (number of SSC, time and duration of the first SSC procedure, and number of hours parents were present and number of caring procedures in which they participated). All data were extracted from the medical records and the nursing flow surveillance sheets, where they were reported as part of standard care.

To evaluate the short-term morbidity and mortality in the study population, data were extracted from the prospective database of the neonatal care department. For all infants, survival without bronchopulmonary dysplasia, defined as no oxygen supplementation or no ventilatory support requirements at 36 weeks post-menstrual age, was determined. For EPIs who survived and completed all hospitalization at Strasbourg University Hospital, the following parameters were analyzed: duration of hospital stay, duration of mechanical ventilation, age at first extubation, duration of non-invasive ventilation, duration of oxygen supplementation, use of post-natal steroids for respiratory support, duration of parenteral nutrition, incidence of necrotizing enterocolitis, incidence of late-onset neonatal sepsis, neurosensory impairment [retinopathy of prematurity (ROP) requiring surgical treatment], intraventricular hemorrhage (IVH), periventricular leukomalacia (PVLM), and post-menstrual age and weight at discharge.

### Statistical Analysis

#### Population Description

Quantitative data were presented as means and standard deviations or medians and ranges (as appropriate) for each study period. Differences were described using ANOVA. Qualitative data were presented as numbers and proportions, and differences were analyzed using the Chi-square test or Fisher's exact test, as appropriate.

#### Outcomes and NIDCAP Criteria

Population characteristics, outcomes, and NIDCAP implementation criteria were described separately for each study period. Comparisons of the first period (2007–2008) and the third period (2013–2014) were performed using linear or logistic regression, as appropriate, with adjustment for GA.

During the second and third periods, survival without broncho-pulmonary dysplasia for neonates who benefited from a NIDCAP observation or intervention and those who did not was compared using logistic regression that adjusted for GA at birth. Odds ratios and 95% confidence intervals (CIs) were presented.

Duration of mechanical ventilation, duration of hospitalization, and weight at discharge were compared using a linear model that adjusted for GA at birth. Linear regression coefficients and 95% CIs were presented.

Data management and analysis were conducted using R software version 3.6.3 (2020, 02, 29).

### Ethical Considerations

This study was approved by the Ethical Committee of the Strasbourg University Medical Faculty and the Institutional Review Board. All parents provided written informed consent for their infants to participate in the prospective recording of medical data in the hospital's database of the unit which was registered at the National Commission on Informatics and Liberty (CNIL) of France.

## Results

### Study Population

**Figure 1:**
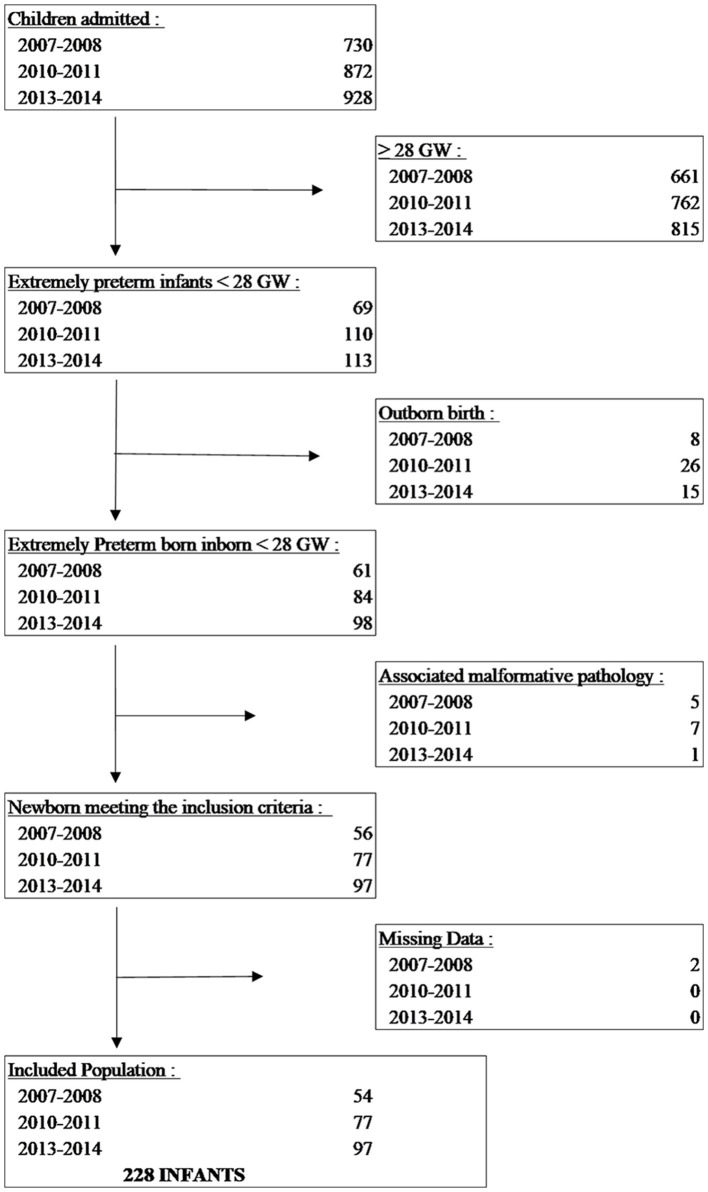
Identification and enrolment of extremely preterm infants.

A total of 2,530 children were admitted to the NICU of the Strasbourg University Hospital during the three study periods, and 292 of them were classified as EPIs, out of whom 242 were inborn. A total of 230 EPIs met the inclusion criteria (Figure 1). Two infants were initially excluded due to missing data. In addition, 10 infants were excluded due to congenital heart defects, 2 were excluded due to intestinal malformations, and 1 was excluded due to spinal muscular atrophy.

The population was significantly more premature during the third period (2013–2014) than the first period (2007–2008), but there were no other significant differences in the three groups (Table 1). Importantly, there were no significant differences in the etiology of preterm birth.

**Table 1:** Characteristics of extremely preterm infants during each study period.

**Characteristic**		**2007–2008 (*n* = 54)**	**2010–2011 (*n* = 77)**	**2013–2014 (*n* = 97)**	***p***
Gestational age (weeks), *n* (%)					0.002
	23–24	3 (5.6)	10 (13)	22 (22.7)	
	25	6 (11.1)	16 (20.8)	15 (15.5)	
	26	14 (25.9)	26 (33.8)	36 (37.1)	
	27	31 (57.4)	25 (32.5)	24 (24.7)	
Birth weight (g), mean (*SD*)		823 (202.9)	799 (180.9)	839 (192.8)	0.48
Male, *n* (%)		28 (51.9)	35 (45.5)	40 (41.2)	0.45
Cesarean section, *n* (%)		39 (72.2)	50 (64.9)	61 (62.9)	0.5
Antenatal steroids, *n* (%)					
	At least 1 injection	48 (88.9)	74 (96.1)	86 (88.7)	0.16
	Complete cure	31 (57.4)	54 (70.1)	54 (55.7)	0.12
CRIB II Score^*^		10.9 (2.4)	11.1 (2.6)	11.5 (2.8)	0.23
Prematurity cause, *n* (%)					
	Vascular	16 (29.6)	13 (16.9)	19 (19.6)	0.1
	Inflammatory	20 (37)	35 (45.5)	54 (55.7)	
	Other	18 (33.3)	29 (37.7)	24 (24.7)	
Death, *n* (%)					
	Withdrawal or withholding of treatment	3 (5.6)	6 (7.8)	12 (12.4)	0.39
	Natural	9 (16.7)	12 (15.6)	10 (10.3)	0.45

* *CRIB II, Critical Risk Index for Babies II*.

### Infant- and Family-Centered Developmental Care Strategies

**Figure 2:**
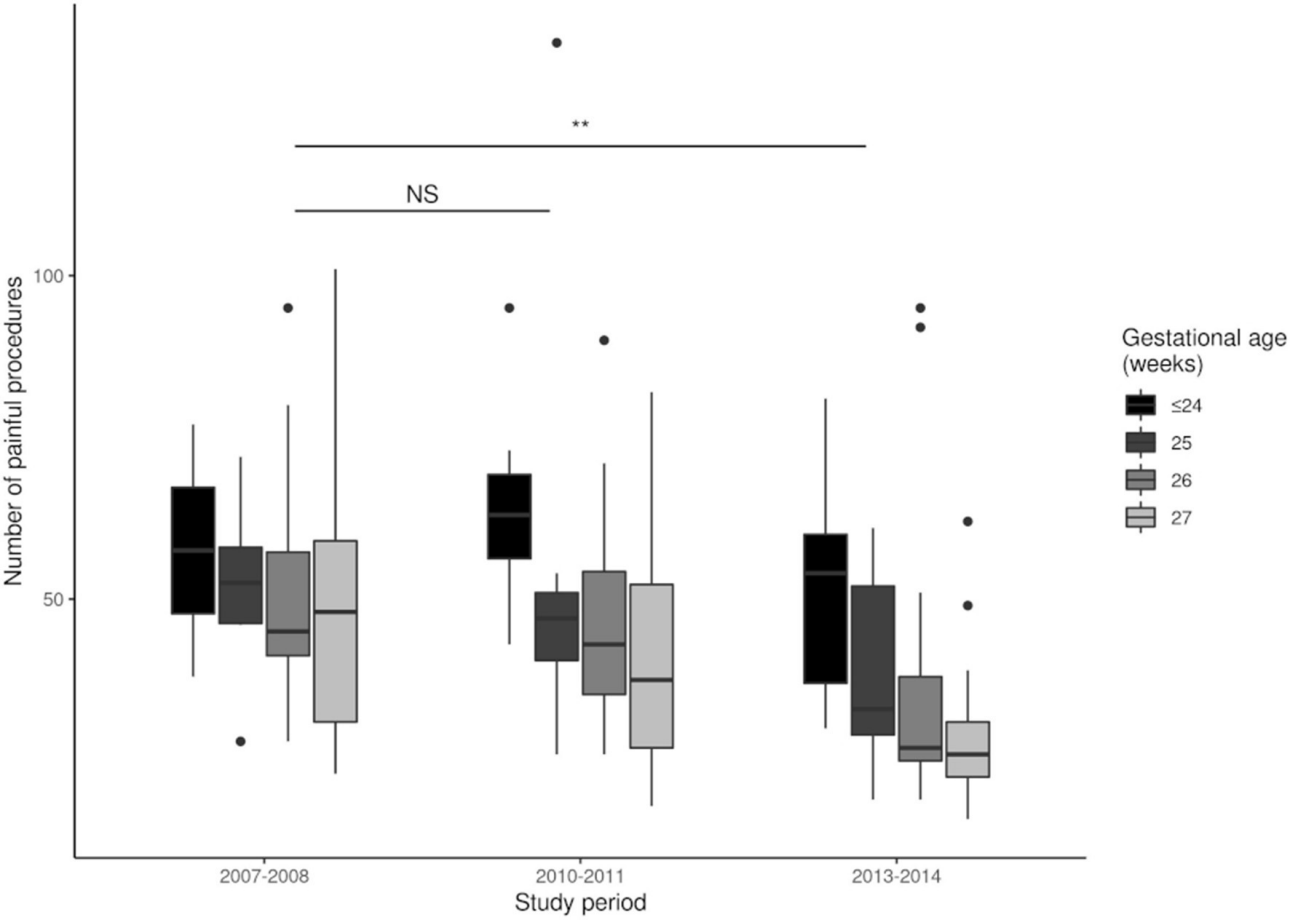
Number of painful procedures during each study period. **p < 0.01; NS, not significant.

**Figure 3:**
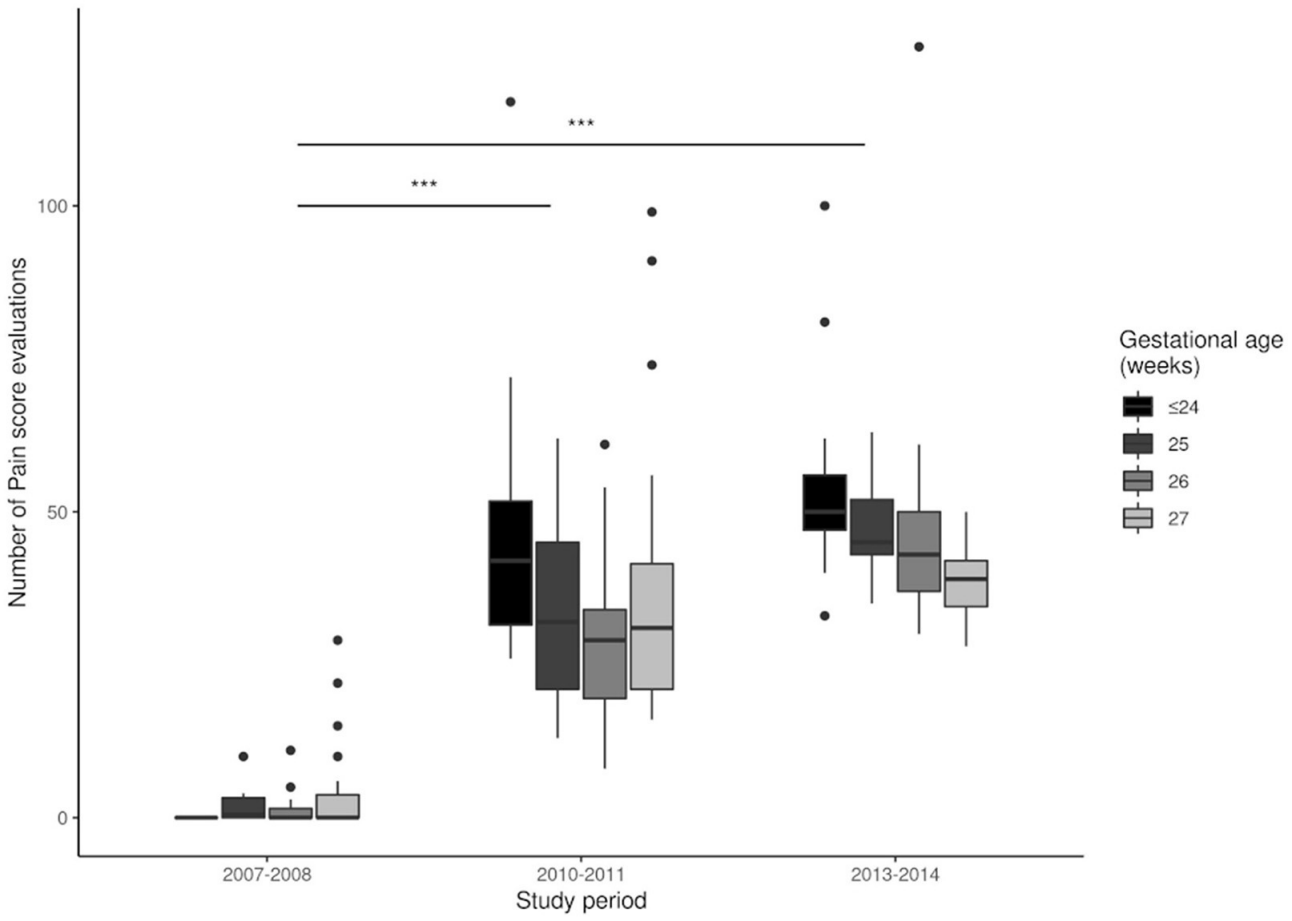
Number of pain score evaluations during each study period. ***p < 0.001.

**Figure 4:**
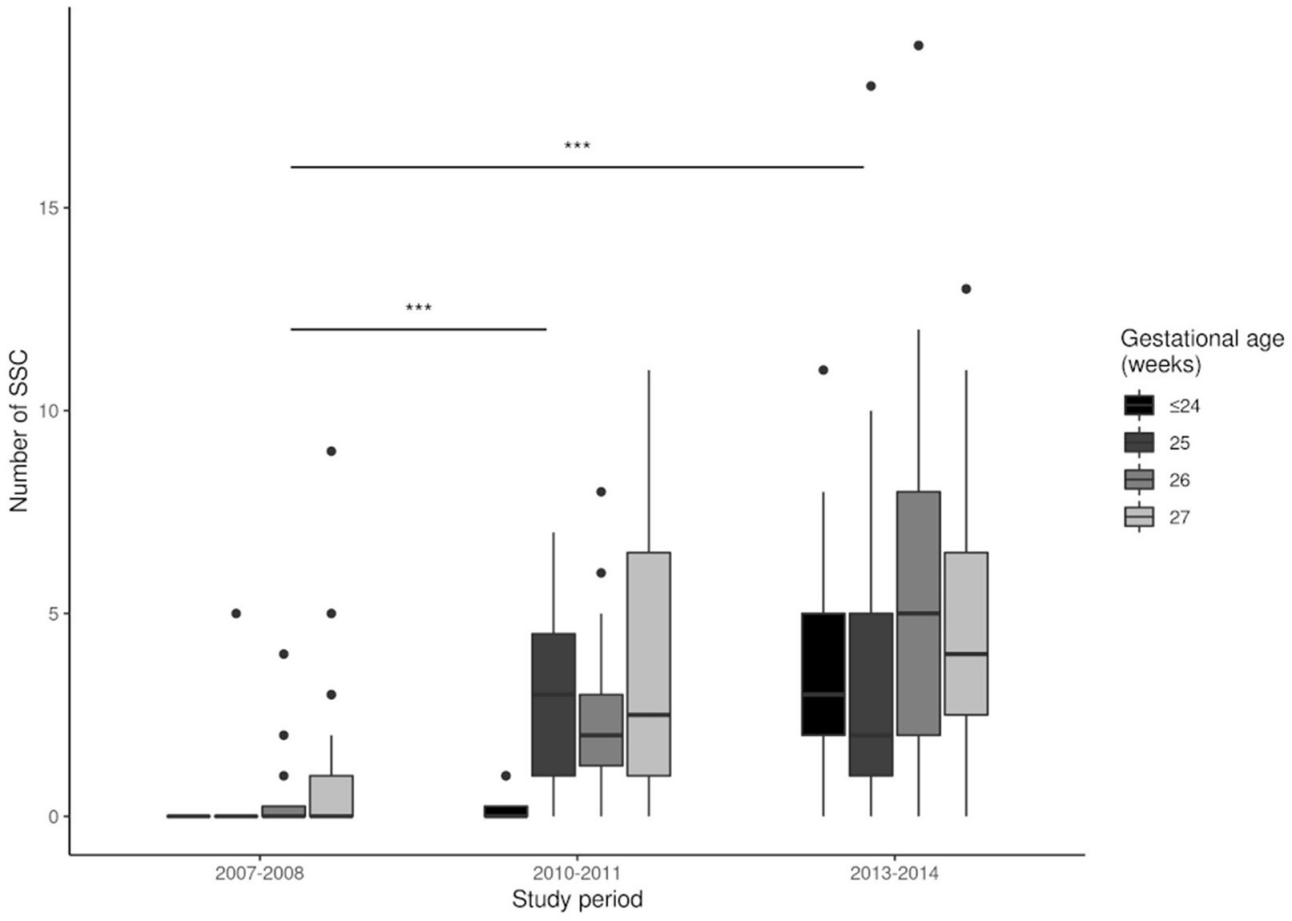
Number of skin-to-skin contact (SSC) events during each study period. ***p < 0.001.

**Figure 5:**
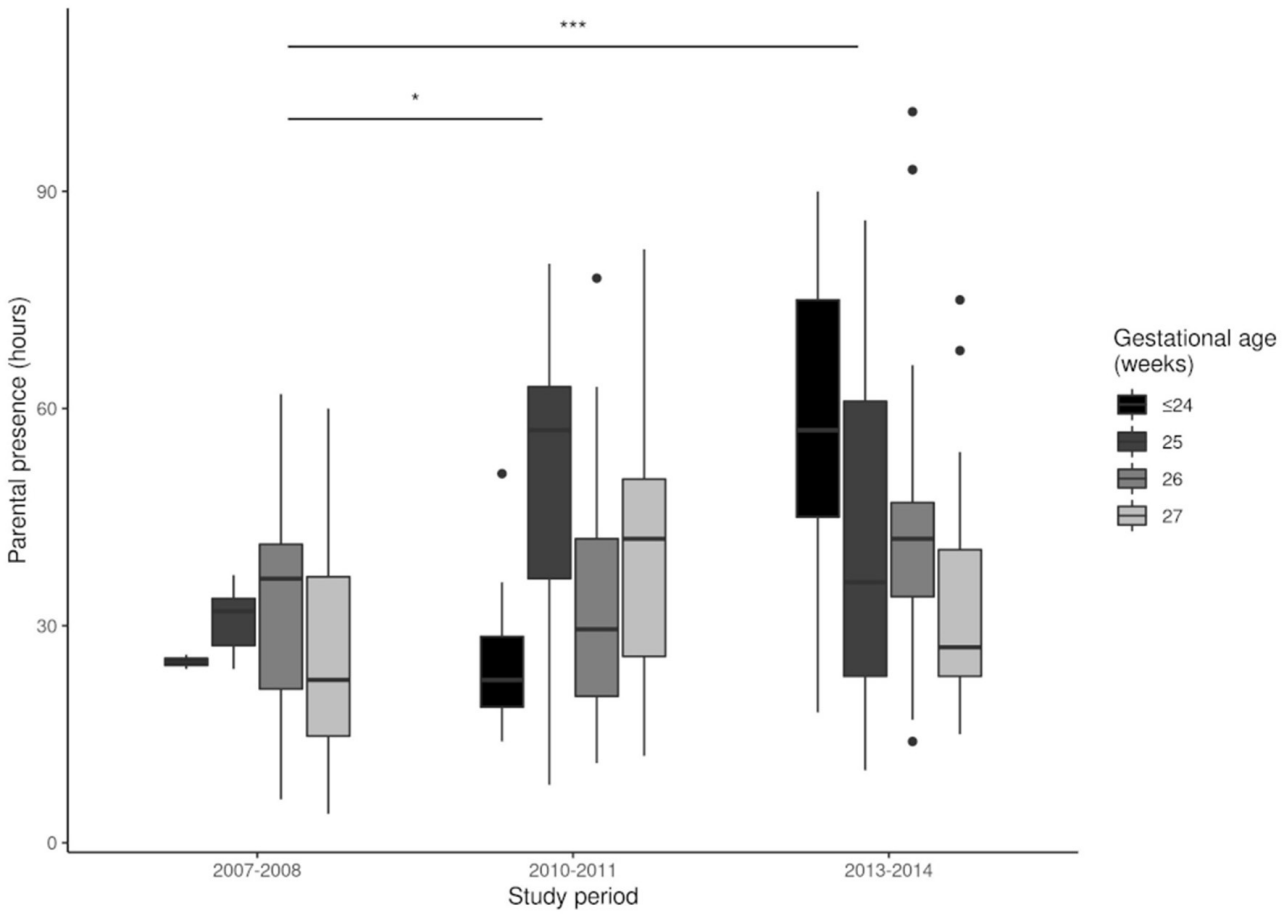
Parental presence during each period. *p < 0.05, ***p < 0.001.

We analyzed the effect of NIDCAP on the implementation of IFCDC practices by comparing the three groups (Table 2). There was a significant increase in the number of NIDCAP observations and in the number of infants who benefited from NIDCAP follow-ups (at least 2 observations). Between 2007 and 2014, there was a significant decrease in the number of painful procedures and an increase in the number of pain assessments (Figures 2, 3). Over time, the amount of SSC increased significantly (Figure 4) and the first SSC was performed significantly earlier and lasted longer. The duration of parental presence and the number of caring procedures to which the parents contributed increased significantly over time (Figure 5).

**Table 2:** Infant and family centered care strategies used in the two first weeks after infant birth during each study period.

**Care strategy**		**2007–2008**	**2010–2011**	**2013–2014**	***p***
		**(*n* = 48)**	**(*n* = 65)**	**(*n* = 86)**	
**NIDCAP**					
	NIDCAP observations, median (range)	0 (0,0)	0 (0,6)	0 (0,8)	<0.001
	At least 1 NIDCAP observation, *n* (%)	0 (0)	9 (13.8)	34 (39.5)	<0.001
	At least 2 NIDCAP observations, *n* (%)	0 (0)	3 (4.6)	26 (30.2)	<0.001
**Pain management**					
	Painful procedures, mean (SD)	49.9 (19.8)	47.5 (20.1)	36.5 (17)	0.001
	Pain Score Evaluation, mean (SD)	2.8 (6)	36 (20.7)	45.8 (13.9)	<0.001
**SSC**					
	At least once, *n* (%)	12 (25)	48 (73.8)	75 (87.2)	<0.001
	If any,				
	Age at first (days), median (range)	9 (4,14)	7 (2,13)	5 (2,13)	0.002
	Duration of first (min), median (range)	60 (60,150)	75 (30,240)	120 (30,300)	0.019
	Sessions, median (range)	2.5 (1,9)	3 (1,11)	5 (1,19)	<0.001
**Parental involvement, median (range)**					
	Presence (h)	26 (4,62)	36 (8,82)	40 (10,101)	0.002
	Participation in care-giving activities	2 (0,10)	10 (0,36)	9 (0,31)	<0.001

*NIDCAP, Neonatal Individualized Developmental Care and Assessment Program; SSC, skin-to-skin contact*.

### Short-Term Medical Outcomes

**Figure 6:**
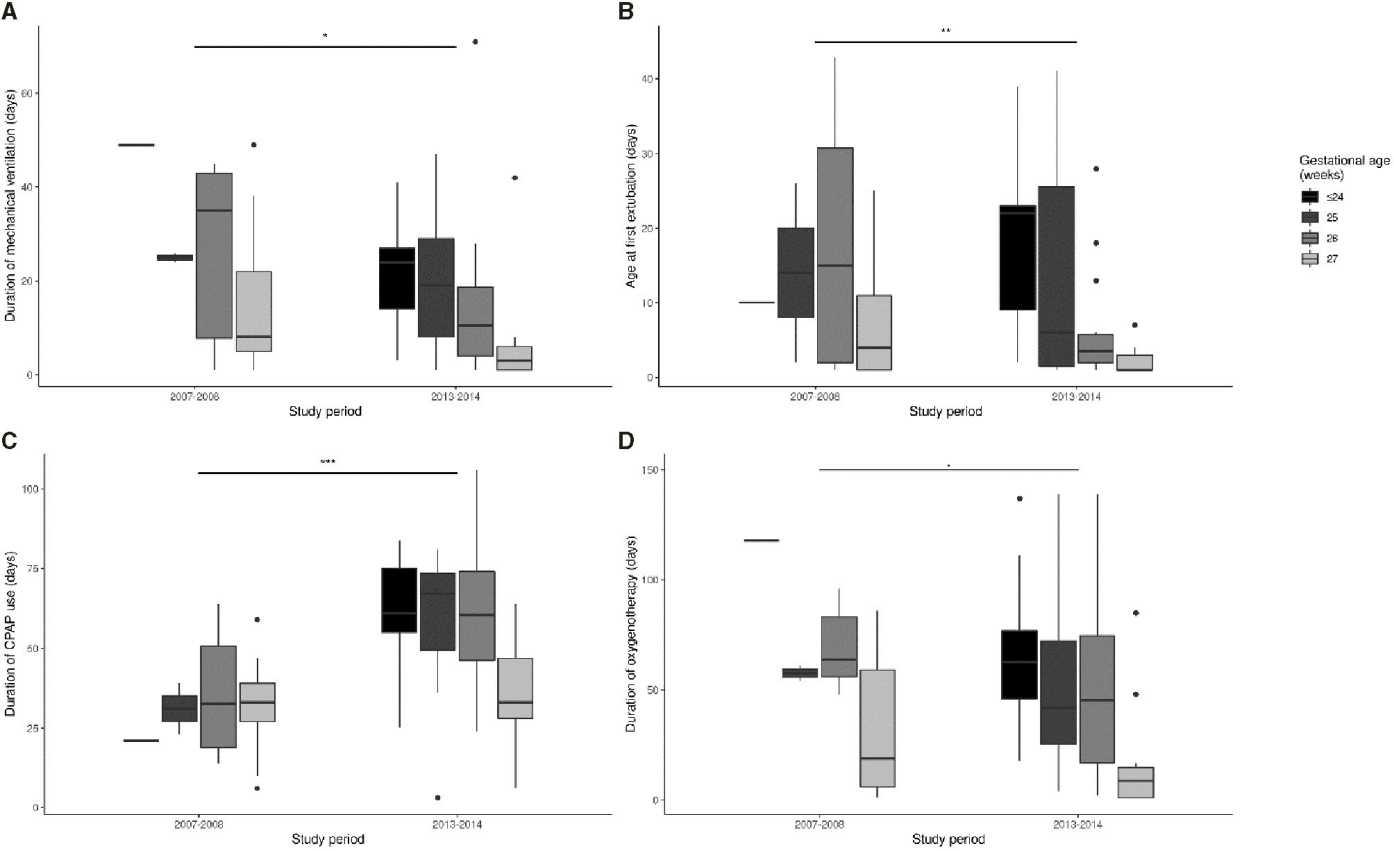
Time of mechanical ventilation **(A)**, from birth to the first extubation **(B)**, CPAP ventilation **(C)**, and oxygen supplementation **(D)** during each study period. *p < 0.05, **p < 0.01, ***p < 0.001.

**Figure 7:**
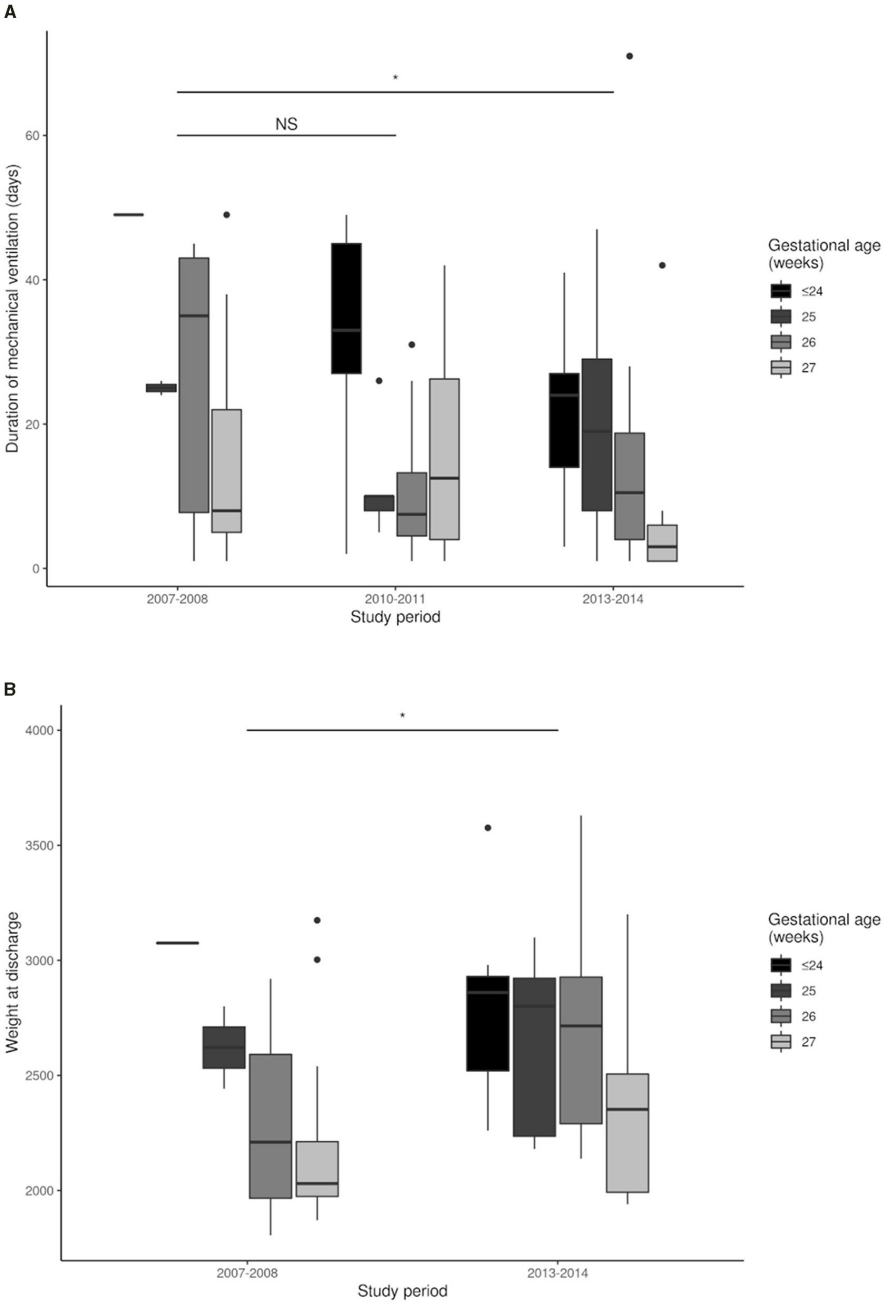
Duration of hospital stay **(A)** and weight gain at discharge **(B)** during each study period. *p < 0.05; NS, not significant.

There were no significant differences in survival and survival without broncho-pulmonary dysplasia during the three study periods after adjustment for GA. A comparison of the short-term outcomes of infants hospitalized until discharge home between the period 2007–2008 and the period 2013–2014 indicated there were significant reductions in the duration of mechanical ventilation (Figure 6A) and in the time from birth to first extubation (Figure 6B), and a significant increase of weight gain at discharge (Figure 7B and Table 3). There was also a significant increase in the duration of CPAP ventilation (Figure 6C). However, the three groups had no significant differences in duration of hospital stay (Figure 7A), duration of oxygen supplementation (Figure 6D), use of postnatal steroids, ROP, IVH, PVLM, late onset neonatal infection, or duration of parenteral nutrition (Table 3).

**Table 3:** Short-term outcomes of infants hospitalized until discharge home at the University hospital of Strasbourg, during each study period, with adjustment for gestational age.

**Outcome**		**2007–2008**	**2010-2011**	**2013-2014**	P^*^
		**(*n* = 34)**	**(*n* = 42)**	**(*n* = 66)**	
**Duration of hospitalization (days), median (range)**		84 (24,154)	94 (15,172)	83 (15,148)	0.97
**Respiratory outcomes (days), median (range)**					
Duration of mechanical ventilation		19 (1,51)	12.5 (1,49)	11.5 (1,74)	0.02
Age at extubation		6 (1,43)	6 (1,43)	3 (1–41)	0.02
Duration of CPAP		29.5 (0,64)	48 (0,75)	53 (0–106)	0.001
Duration of 0_2_		53.5 (1,118)	36 (1,172)	27.5 (1–140)	0.25
**Neurological outcomes**, ***n*****(%)**					
ROP		2 (5.9)	6 (14.3)	11 (16.7)	0.14
PVLM		2 (5.9)	0 (0)	8 (12.5)	0.31
IVH	None	29 (85.3)	23 (54.8)	45 (68.2)	0.15
	Grade 1,2	3 (8.8)	15 (35.7)	17 (25.8)	0.09
	Grade 3,4	2 (5.9)	4 (9.5)	4 (6.1)	0.75
**NEC**, ***n*****(%)**		5 (14.7)	10 (23.8)	13 (19.7)	0.53
**Infection**, ***n*****(%)**					
Bacterial LONI	None	6 (17.6)	7 (16.7)	13 (19.7)	0.22
	Probable	8 (23.5)	7 (16.7)	15 (22.7)	0.96
	Certain	20 (58.8)	28 (66.7)	38 (57.6)	0.39
Fungal LONI		5 (14.7)	7 (16.7)	16 (24.2)	0.27
**Age at full enteral feeding (days), median (range)**		45 (31,150)	49 (23,130)	46 (18,116)	0.09
**Weight at discharge (g), mean (SD)**		2276 (412)	2634 (598)	2635 (422)	0.02

* *Comparison of the first period (2007–2008) and third period (2013–2014).*

*LONI, late onset neonatal infection; NEC, necrotizing enterocolitis; ROP, retinopathy of prematurity; IVH, intraventricular hemorrhage; PVLM, periventricular leukomalacia*.

**Figure 8:**
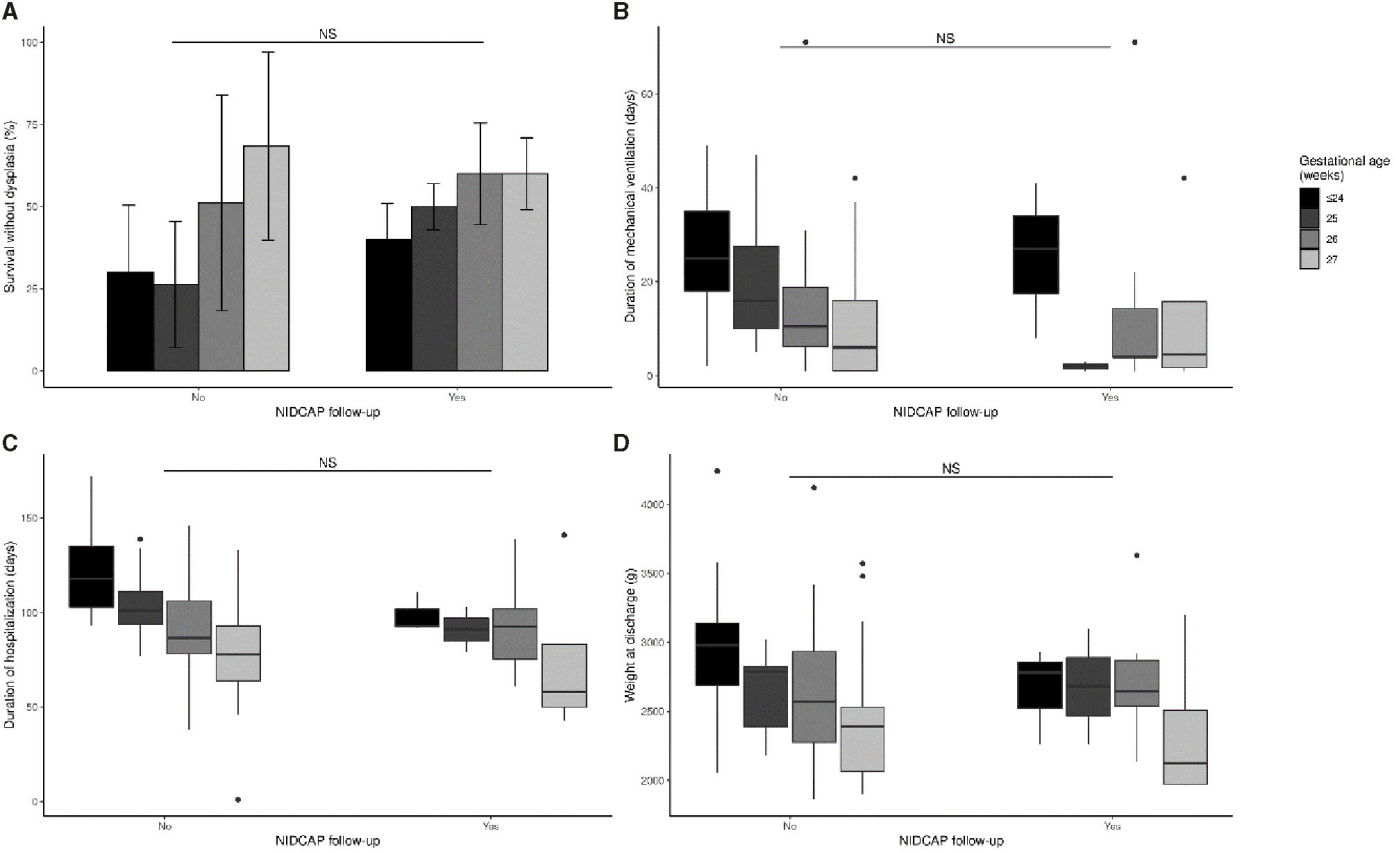
Impact of NIDCAP intervention on survival without dysplasia **(A)**, duration of mechanical ventilation **(B)**, duration of hospitalization **(C)**, and weight gain at discharge **(D)**. NS, not significant.

The NIDCAP intervention (with at least 2 observations during the hospital follow-up), during the second and third periods and adjusted for GA, was not significantly associated with survival without broncho-pulmonary dysplasia, duration of mechanical ventilation, duration of hospitalization, and weight at discharge (Figures 8A–D).

## Discussion

The major result of this observational study is that the NIDCAP was effective in promoting measurable improvements in IFCDC practices at a level III regional NICU during the implementation process, in the population of EPI. This improvement in IFCDC practices was also temporally associated with moderate but measurable improvements in the short-term medical outcomes of EPIs.

This study had some limitations. It was a single center retrospective study of a relatively small number of infants. However, all the IFCDC data were fully available on the infants' charts and were reported using standard care procedures. The outcome data were also prospectively recorded for each infant supporting their validity. Previous researchers highlighted the importance of observational studies, such as the present study, for assessment of the implementation of all components of a complex intervention, because comprehensive assessments can be difficult in RCTs (19, 20). This is particularly the case for IFCDC, because there are specific barriers that preclude full evaluation (25).

### NIDCAP Observations and Implementation

The implementation of the NIDCAP was associated with a significant increase in IFCDC practices. As expected, these changes were temporally associated with an increased number of NIDCAP observations, demonstrating the progressive implementation of individualized developmental care in the NICU. NIDCAP observations, which are performed by NIDCAP-certified professionals, are the cornerstone of this program (26, 27). However, we found that only a minority of EPIs benefited from NIDCAP observations, even during the third observation period.

### Infant Pain Management

The NIDCAP led to a significant decrease in the number of painful procedures and an increase in the evaluation of pain scores. Due to study design, we were unable to directly evaluate pain scores in the whole study population. However, because painful procedures are the main causes of pain in the NICU (28), our data suggest there were improvements in the well-being of EPIs during the NIDCAP implementation. This is in accordance with previous reports of the beneficial impact of IFCDC on pain management. Indeed, infant pain management is an important component of IFCDC, and previous studies assessed pain to evaluate the implementation and the short- and long-term benefits of developmental care programs (29–31).

This holistic approach that uses environmental strategies based on individual evaluation of an infant's behavior and needs can reduce infant pain due to an increased number of close observations of the infant, high parental involvement as primary caregivers, and coordinated use of non-pharmacological treatments that provide pain relief. The precise and individualized evaluation of signs of withdrawal and approach of each child during NIDCAP observations may allow healthcare providers to individualize caring procedures, adjust their duration according to the tolerance of each child, and promote grasping opportunities that support autoregulation of the infant. Previous studies used NIDCAP cues to evaluate pain and integrated this into different pain scores (32–34).

Increased parental presence can improve pain management of infants, and is associated with lower pain scores. For example, the large EPIPPAIN 2 study reported that pain scores following venipuncture of newborns were lower when the parents were present (35). Other studies reported that IFCDC strategies effectively reduced pain during and after routine caring procedures (36, 37). A prospective observational study in the Netherlands reported that the implementation of NIDCAP-based stress reduction strategies which included infant pain management significantly reduced the number of painful interventions in the NICU (38). Another study reported that the NIDCAP by itself effectively decreased stress- and pain-related behaviors, physiological stress responses, and the use of sedatives and opioids in neonates (39).

### Parental Presence, SSC, and Participation in Care

The NIDCAP led to significantly more presence and involvement of parents in the care of their infants, in that they had earlier, more prolonged, and an increased number of SSC procedures. Similarly, the EPIPAGE 2 study in France showed that the unit policies and the presence of a formalized developmental care program were significantly associated with an earlier initiation of SSC, which had no limitation of duration (24). Our study provided detailed data that confirmed this finding at the level of an individual center. A study in Sweden, which has active promotion of IFCDC practices, reported the average initiation of SSC for EPIs was on the sixth day of life in seven regional hospitals during 2011 (40). Longer durations of parental involvement and increased SSC were reported in countries of Northern Europe, suggesting potential progress in our unit as compared to these countries (41, 42). There is also evidence that the full integration of both parents in the care of their children in family rooms inside neonatal units, from admission until discharge, provided medical benefits to the infants, in that it significantly decreased the duration of hospital stay and the incidence of broncho-pulmonary dysplasia (18). European guidelines thus recommend provision of family rooms, including in the NICU, where the EPIs are hospitalized during their first 14 days of life (11, 43).

### Short-Term Medical Outcomes

A secondary aim of this study was to determine whether implementation of the NIDCAP as an effort to increase IFCDC practices was already, despite incomplete implementation, temporally associated with benefits in the short-term medical outcomes of the children who, however, were significantly more immature overtime. Indeed, over time we observed an increased number of EPIs who were cared for in the NICU and a decrease in the gestational age of EPIs. We observed no significant changes in mortality or survival free of broncho-pulmonary dysplasia, but we found a significant decrease in the duration of mechanical ventilation, earlier first extubation, and a significant increase in body weight at discharge. We also found a significant increase in the duration of non-mechanical ventilation and a non-significant decrease in the duration of oxygen therapy. This decrease in invasive respiratory support during NIDCAP implementation is consistent with the results of a Cochrane review that evaluated developmental care (13) and of several RCTs that evaluated the NIDCAP (15, 27, 44). However, these positive results regarding respiratory support might also be due to changes in the management of respiratory distress syndrome between 2007 and 2014, as illustrated by the evolution of international recommendations (45–47). In our unit, guidelines are regularly updated to reduce the duration of mechanical ventilation, to support the increased use of non-invasive respiratory support, and to promote less-invasive surfactant administration. We also cannot totally exclude that the significant increase in body weight at discharge might be due to changes in nutritional practices, in addition to changes in IFCDC practices. However, previous research also reported that NIDCAP was associated with an increased daily weight gain (14). Moreover, increased parental involvement could also explain the increased weight gain of these infants. A Canadian multicenter, cluster randomized trial which compared standard care to Family Integrated Care (FICare; a family-centered care program with a parental involvement of at least 6 h per day) found that FICare led to a significant increase in the weight of very preterm infants at day 21. However, there were no reported benefits of FICare on infant mortality and short term morbidity (48).

Our finding that implementation of the NIDCAP did not affect the duration of hospitalization or the incidence of bronchopulmonary dysplasia was not in accordance with previous studies. In particular, these benefits were reported in a meta-analysis of well-designed and adequately powered RCTs of NIDCAP whose specific aims were to evaluate these outcomes (14). Our contrary finding may be because only about one-third of our infants benefited from at least one NIDCAP observation during the last period. Incomplete NIDCAP implementation or NIDCAP-based care that is provided by NIDCAP-educated staff members and behavioral observations that are interrupted before discharge were previously blamed for the discrepant results of two RCTs of NIDCAP (15, 16). In particular, the study by Maguire et al. showed no measurable short-term benefit from the program, but nearly 50% of children were transferred out of the NIDCAP center before hospital discharge (16). In contrast, Peters et al. identified short- and medium-term benefits for children who received the full and continuous hospital NIDCAP-based care (15, 49). In the present observational study, our comparison of periods 2 and 3 indicated no significant differences between EPIs who received NIDCAP-based care (*n* = 29) and whose who did not (*n* = 151), although there was a trend of decreased duration of mechanical ventilation and length of hospital stay in the NIDCAP group. This may be due to a lack of statistical power from our small sample size. Another possible reason for our negative findings regarding the effect of NIDCAP on the duration of hospitalization and the incidence of bronchopulmonary dysplasia may be that NIDCAP-based care requires application of the recommendations for the care of infants based on NIDCAP observations by NIDCAP-certified professionals. This implies a sufficient level of knowledge and implementation of IFCDC strategies by most caregivers who are working in the NICU.

### Monitoring of IFCDC Practices and NIDCAP Implementation

The implementation of the NIDCAP takes time, as indicated by the progressive improvements in IFCDC practices observed in the present study, and it also requires changes in the hospital system. Thus, the NIDCAP is a complex intervention whose implementation may be challenging (21, 22). Monitoring of implementation of IFCDC practices, as performed in this study, is an approach that can support the efforts of caregivers. This monitoring should be continued and used as a standard for assessment of the quality of care in all neonatal units (29) because it can lead to a better neurodevelopmental outcomes of preterm infants.

At a clinical level, individualization of care should be used during the NIDCAP implementation for most infants. Training supported by the same theoretical bases at an intermediate level is available to facilitate the dissemination of NIDCAP-based care within a neonatal department and within a perinatal network where infants are transferred back from a level III referral center. Training to the Family & Infant Neuro-Development Education (FINE) (50) or the Support of Oral Feeding in Fragile Infants (SOFFI) (51) programs are more accessible for neonatal centers outside level III, and could facilitate the continuity of infant care in a perinatal network.

Although a systematic review of RCTs that examined NIDCAP found no evidence that this program improved long-term neurodevelopmental outcomes, these authors reported that NIDCAP was associated with several short-term medical benefits, including shorter hospitalization and increased Bayley Scale of Infant Development scores at 9–12 months (14). Many studies showed that improvements of infant pain management, early SSC, and parental involvement had long lasting positive effects on the neurodevelopment of preterm infants (29–31, 52–54). Because NIDCAP significantly increases the implementation of IFCDC practices, it may also provide long-term neurodevelopmental benefits, in addition to the well-documented short-term benefits.

## Conclusion

Our results indicated that implementation of the NIDCAP was associated with a greater use of IFCDC practices that led to prevention of pain, increased parental involvement in the care of children, and increased SSC of parents and children. These changes occurred rapidly and were readily measurable during implementation of this program. It is essential to employ more long-term monitoring of changes in IFCDC practices to assess improvements in the quality of care delivered to these vulnerable newborn infants. Research that assesses the implementation of a complex intervention such as the NIDCAP may provide a better understanding of the efficacy of different specific practices and support their inclusion in daily practice. The effect of these changes on the developmental trajectories of EPIs needs further evaluation.

## Data Availability Statement

The raw data supporting the conclusions of this article will be made available by the authors, without undue reservation.

## Ethics Statement

The studies involving human participants were reviewed and approved by Comité d'Éthique de la Faculté de Medicine de Strasbourg. Written informed consent to participate in this study was provided by the participants' legal guardian/next of kin.

## Collaborative Authors

Strasbourg NIDCAP Study group: Jamal Beladdale, Caroline Briot, Céline Cayeux, Gwenaelle Fourie, Florence Gehant, Martine Hausser, Audrey Huffschmidt, Sylvie Kracher, Antoine Martenot, Jacqueline Matis, Solange Mellado, Oscar Monroy, Stéphanie Poirot, Jennifer Rondel, Christine Scheib and Isabelle Zimmer.

## Author Contributions

VK participated in the design of the study, was the main contributor of data extraction and analysis, literature review, and wrote the first draft of the manuscript. CZ-K, LD, CL, BE, and DA discussed the study design, contributed to the data collection, and the writing of the manuscript. They all approved the final version of the manuscript. IL performed the statistical analysis, generated the figures, contributed to the writing of the manuscript, and reviewed the last version of the manuscript. PK was the main coordinator of the study design, data analysis, and writing of the manuscript, he thoroughly reviewed the last version of the manuscript. All authors contributed to the article and approved the submitted version.

## Conflict of Interest

The authors declare that the research was conducted in the absence of any commercial or financial relationships that could be construed as a potential conflict of interest.

## Publisher's Note

All claims expressed in this article are solely those of the authors and do not necessarily represent those of their affiliated organizations, or those of the publisher, the editors and the reviewers. Any product that may be evaluated in this article, or claim that may be made by its manufacturer, is not guaranteed or endorsed by the publisher.

## Abbreviations

EPI: extremely preterm infants
IFCDC: infant and family centered developmental care
NIDCAP: Neonatal Individualized Developmental Care and Assessment Program
SSC: skin-to-skin contact.
